# When I say … reciprocal illumination

**DOI:** 10.1111/medu.12743

**Published:** 2015-08-22

**Authors:** Roger Kneebone

**Affiliations:** London, UK

This paper proposes the idea of education as *engagement* rather than transmission, by which I mean an open-minded exchange of perspectives resulting in ‘reciprocal illumination’ for everyone who takes part. Terminology is important here. Mention the term ‘public engagement’ and people think of experts pontificating about their pet subject – at science fairs, perhaps, or outreach activities. The assumption is that experts know lots and transmit their knowledge without being changed much, and that members of the public don't know much at all and stand to gain far more. However, perhaps a different framing might open new opportunities.

This takes us back to how we learn. From primary school classroom to university lecture theatre, the classic model is ingrained in us all. The very word ‘education’ transports us to the schoolroom: we are sitting at a little desk, perhaps, or teaching in front of a class. At the heart of this model is an assumption that education is a one-way process, whereby learners learn but teachers don't. Of course all experienced teachers know this isn't true; in reality, we learn from our students all the time. Yet the educational system is heavily weighted towards one-way traffic. And the idea of education as transmission remains deeply ingrained.

This paper puts forward an alternative: that education is primarily about interaction. If we frame this as a process in which *everyone* expects to gain new insights – the ‘reciprocal illumination’ referred to in the title – new possibilities arise. This is especially relevant in medicine, with its inbuilt power gradients. As clinicians, we spend a lot of time conveying information, but much less taking it in. We seldom get the chance to see ourselves as others see us, and even less as our patients see us. Yet this can be the most useful insight of all.

Of course, people have been talking about this for decades. Nonetheless, the transmission model remains remarkably robust, and is alive and kicking today. Not that there's anything wrong with transmission *per se* – there are plenty of times when it's highly appropriate.^1^ Problems emerge when it becomes the dominant mode, when educational activity in general excludes reciprocity.

This model is rooted in the 19th and 20th centuries, with their structures of deference and authority. However, the world works differently now.^2^ People gather and exchange information, perspectives and viewpoints in other ways – through Wikipedia, social media, impromptu conversations. It's about exchange, not transmission.

So how might we achieve this kind of interaction, whether as teachers, learners, patients or clinicians? How might we minimise the power differentials that permeate those traditional ways? Perhaps a different, older model might serve us better than the schoolroom. Richard Sennett talks about the ‘early modern’ (Renaissance) workshop like this:

‘The precept ruling the early modern workshop was that informal, open-ended cooperation is how best to experience difference. Each of the terms in this precept matters. “Informal” means that contacts between people of different skills or interests are rich when messy, weak when they become regulated, like boring meetings run strictly on formal rules of order. “Open-ended” means you want to find out what another person is about without knowing where it will lead; put another way, you want to avoid the iron rule of utility that establishes a fixed goal – a product, a policy objective – in advance. “Cooperation” is the simplest and most important term. You suppose that different parties all gain by exchanging, rather than one party gaining at the expense of others.’^3^

This sharing of perspectives with the intention of changing all concerned is difficult to achieve. Yet perhaps this could be the model for a different kind of education – a kind that fits with our social world, not one that pushes against it. Rather than ‘*public* engagement’, perhaps we can use the simpler term ‘engagement’ to crystallise a different dynamic. By framing engagement as interaction – between some people with specialist expertise and other people with different (although equally valid) expert perspectives – new understandings are brought into focus and fresh insights emerge.

This is not to devalue expert knowledge – far from it. Yet by identifying expertise as a part of the picture – rather than the whole of it – we allow different ways of seeing to unfold. By reframing engagement as a generous exchange of perspectives aimed at reciprocal illumination, perhaps we can rethink what we mean by education.
